# Pathogenesis of *CDK8-*associated disorder: two patients with novel *CDK8* variants and in vitro and in vivo functional analyses of the variants

**DOI:** 10.1038/s41598-020-74642-4

**Published:** 2020-10-16

**Authors:** Tomoko Uehara, Kota Abe, Masayuki Oginuma, Shizuka Ishitani, Hiroshi Yoshihashi, Nobuhiko Okamoto, Toshiki Takenouchi, Kenjiro Kosaki, Tohru Ishitani

**Affiliations:** 1 Center for Medical Genetics, Keio University Hospital, Tokyo, Japan; 2 Institute for Molecular and Cellular Regulation, Gunma University, Maebashi, Japan; 3 Department of Genetics, Tokyo Metropolitan Children’s Medical Center, Tokyo, Japan; 4 Department of Medical Genetics, Osaka Women’s and Children’s Hospital, Osaka, Japan; 5 Department of Pediatrics, Keio University School of Medicine, Tokyo, Japan; 6 Department of Homeostatic Regulation, Research Institute for Microbial Diseases, Osaka University, 3-1 Yamadaoka, Suita, Osaka 565-0871 Japan

**Keywords:** Disease genetics, Disease model

## Abstract

Cyclin-dependent kinase 8 (CDK8) is a member of the CDK/Cyclin module of the mediator complex. A recent study reported that heterozygous missense *CDK8* mutations cause a neurodevelopmental disorder in humans. The mechanistic basis of *CDK8*-related disorder has yet to be delineated. Here, we report 2 patients with de novo missense mutations within the kinase domain of CDK8 along with the results of in vitro and in vivo functional analyses using a zebrafish model. Patient 1 and Patient 2 had intellectual disabilities and congenital anomalies. Exome analyses showed that patient 1 had a heterozygous de novo missense p.G28A variant in the *CDK8* (NM_001260.3) gene and patient 2 had a heterozygous de novo missense p.N156S variant in the *CDK8* gene. We assessed the pathogenicity of these two variants using cultured-cells and zebrafish model. An in vitro kinase assay of human CDK8 showed that enzymes with a p.G28A or p.N156S substitution showed decreased kinase activity. An in vivo assays of zebrafish overexpression analyses also showed that the p.G28A and p.N156S alleles were hypomorphic alleles. Importantly, the inhibition of CDK8 kinase activity in zebrafish embryos using a specific chemical inhibitor induced craniofacial and heart defects similar to the patients’ phenotype. Taken together, zebrafish studies showed that non-synonymous variants in the kinase domain of *CDK8* act as hypomorphic alleles causing human congenital disorder.

## Introduction

Cyclin-dependent kinase 8 (CDK8) is an evolutionarily conserved serine/threonine kinase that belongs to the CDK/Cyclin module of the mediator complex. CDK8 modulates a variety of transcription regulators, including HIF1A, p53, and NRSF, both positively and negatively^1–3^ and controls signaling pathways, including the WNT/ß-catenin signaling pathway^4^.

**Figure 1:**
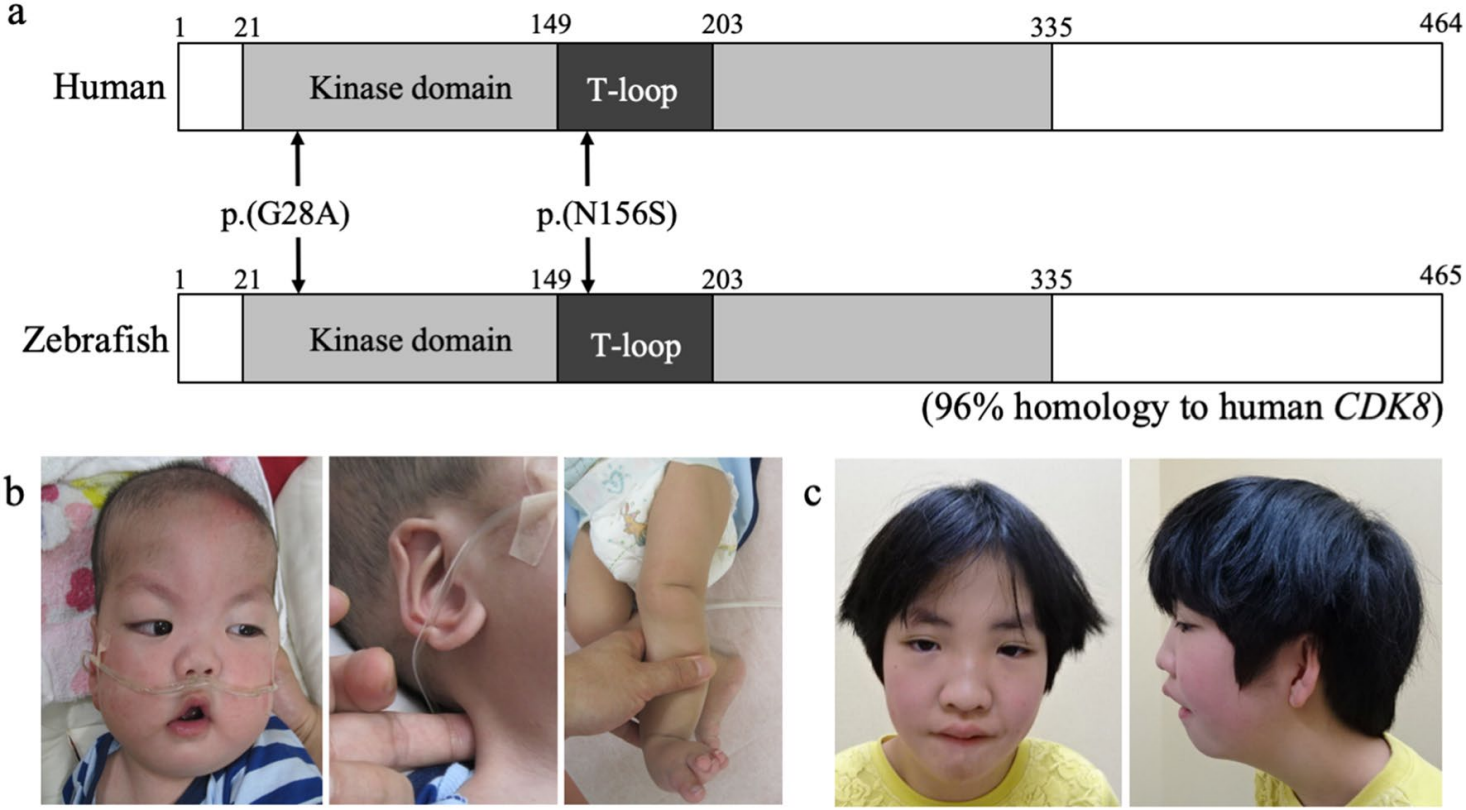
Schema of the CDK8 protein and clinical characteristics of our two patients with CDK8-associated disorder. (**a**) Schema of human CDK8 protein (the upper schema) and zebrafish Cdk8 protein (the lower schema). The grey box indicates the kinase domain. The black boxes indicate the T-loop in the kinase domain. The arrows point to the mutations of the 2 reported patients. (**b**) Patient 1 at 8 months of age. (**c**) Patient 2 at 8 years of age. In 1b, note the prominent forehead with large anterior fontanelle, arched eyebrow, hypertelorism, epicanthal folds, low-set ears, prominent antihelix, short philtrum, flat nasal bridge, high arched palate, down-turned corners of the mouth, micrognathia, webbed neck, and overlapping toes on the left foot. In 1c, note the hypertelorism, epicanthal folds, wide nasal bridge, protruding ears, bifid uvula, and micrognathia.

A recent study reported the cases of 12 patients with eight heterozygous non-synonymous variants in the kinase domain (21–335 amino acids, Fig. 1a)^5^ of the *CDK8* gene who presented with characteristic facial features, congenital heart disorders and intellectual disability^6^. An in vitro functional analysis of the eight types of variants in the kinase domain of CDK8 demonstrated that *CDK8*-associated disorder is caused by hypomorphic alleles of *CDK8*^6^. However, the mechanistic basis of *CDK8*-associated disorder in vivo has yet to be determined. In this article, we report an in vivo functional analysis in a zebrafish model with de novo amino-acid substitutions (p.G28A and N156S) in the kinase domain of CDK8, as detected in 2 patients with syndromic intellectual disability.

## Results

### Clinical features

Patient 1 was a 3-year-old Japanese boy with a birth weight of 2246 g (− 0.4 SD), a body length 46.5 cm (+ 0.1 SD), and a head circumference of 34 cm (+ 1.3 SD) who had been diagnosed at birth with a double outlet right ventricle, mitral valve stenosis, and dysmorphic craniofacial features. A Glenn operation was performed at 7 months of age. The patient had difficulty feeding and failed to thrive. Severe gastroesophageal reflux disease was treated by fundoplication and a gastrostomy. He also had an undescended left testis and left multicystic dysplastic kidney. A brain magnetic resonance imaging study showed agenesis of the corpus callosum. Markedly delayed motor development and language development had been observed since infancy. Generalized hypotonia was noted, and an eye examination revealed myopia.

On physical examination, his body height was 87.3 cm (− 2.1 SD), his body weight was 10.9 kg (− 1.9 SD), and his head circumference was 49.7 cm (+ 0.1 SD). Craniofacial dysmorphic features were observed and are described in Table 1 (Fig. 1b, Table 1); overlapping toes were noted on the left foot (Fig. 1b). Routine laboratory investigations and a metabolic survey yielded results within the normal range. A chromosomal analysis showed a normal male karyotype (46, XY), and array-based comparative genomic hybridization yielded normal results.

**Table 1:** Summary of the patients with neurodevelopmental disorder and heterozygous variants in *CDK8.*

	Patient 1	Patient 2	Calpena et al.^6^	14 patients with CDK8 variants
Variants in CDK8	c.83G > C (p.Gly28Ala)	c.467A > G (p.Asn156Ser)	c.79G > C (p.Val27Leu) c.85C > G (p.Arg29gly) c.88G > A (p.Gly30Ser) c.185C > T (p.Ser62Leu) × 5 c.291T > G (p.Phe97Leu) c.533G > A (p.Arg178Gln) c.578T > G (p.Val193Gly) c.669A > G (p.Ile223Met)	All 14 mutations were missense
Sex	Male	Female	7 males and 5 females	Male: 8, Female: 6
Developmental delay	Severe	Moderate	9/12	11/14
Intellectual disability	Severe	Moderate (WISC-IV IQ = 52)	11/12	13/14
Short stature	Present	Present	1/12	3/14
Hypotonia	Present	Present	9/11	11/13 (1 was not available)
Head and necks	Agenesis of corpus callosum	No abnormalities	Abnormality of corpus callosum: 4/9 (among 9 available patients)	Abnormality of corpus callosum: 5/11 (3 were not available)
Congenital heart disorders	DORV, mitral valve stenosis	Absent	Present: 6/10	7/12 (2 were not available)
Facial dysmorphology	Prominent forehead with large anterior fontanelle, arched eyebrow, hypertelorism, epicanthal folds, low set ears, prominent antihelix, a short philtrum, broad nasal root, a flat nasal bridge, a high arched palate, down turned corners of mouth, micrognathia	Arched eyebrow, ptosis, hypertelorism, epicanthal folds, broad nasal root, anteverted nares, a wide nasal bridge, protruding ears, bified uvula, micrognathia	Arched eyebrow (3/12), epicentral folds (4/12), ptosis (5/12), anteverted nares (3/12), broad nasal root (3/12), micrognathia (4/12)	Arched eyebrow (5/14), ptosis (5/14), epicanthal folds (6/14), broad nasal root (5/14), anteverted nares (5/14), micrognathia 6/14)
Behavioral/psychiatric manifestations	Absent	Tantrum, agitation, emotional incontinence, attention deficit-hyperactivity disorder (ADHD)	ADHD: 4/10, autism spectrum disorder: 5/10	ADHD: 5/12 (2 were not available)
Eye/vision	Myopia	Intermittent esotropia	Myopia: 5/12	Myopia (6/14)
Gastrointestinal problems	Gastroesophageal reflux disorder (GERD)	Absent	GERD: 3/12	GERD (4/14)
Urogenital problems	Left multicystic dysplastic kidney	Absent	0/12	1/14
Limbs	Absent	Pes planovalgus	Pes planovalgus: 2/12	3/14

Patient 2 was an 8-year-old Japanese girl who had been diagnosed with pharyngostenosis and gastroesophageal reflux disease at 6 months of age. Her developmental milestones were delayed. At 8 years, 3 months, her body height was 115.8 cm (− 2 SD), her body weight was 18.3 kg (− 2.5 SD), and her head circumference was 52.0 cm (mean). She exhibited the dysmorphic features described in Table 1 (Fig. 1c, Table 1) as well as intermittent esotropia and pes planovalgus. At 8 years of age, her developmental quotient as assessed using the WISC-IV test was 52. She also exhibited tantrums, hyperactivity, agitation, emotional incontinence, and attention deficit.

### Next-generation sequencing

An exome analysis of genomic DNA derived from peripheral blood samples from Patient 1 and Patient 2 showed a constitutional heterozygous de novo non-synonymous variant in exon 1 of the *CDK8* gene (NM_001260.3): c.83G > C, (p.Gly28Ala) and in exon 5 of the *CDK8* gene: c.467A > G, (p.Asn156Ser). We confirmed these findings using Sanger sequencing. The combined annotation-dependent depletion (CADD) scores^7^ for p.Gly28Ala and p.Asn156Ser were 23.9 and 26.2, respectively. The amino acid substitutions in CDK8 detected in these 2 patients were within the ATP-binding domain of the kinase domain of CDK8 protein (Fig. 1a)^5^. Neither of these variants was found in the normal 2048 Japanese database^8^ or in the ExAC database^9^.

### In vitro kinase assay of human CDK8 containing G28A and N156S substitutions

**Figure 2:**
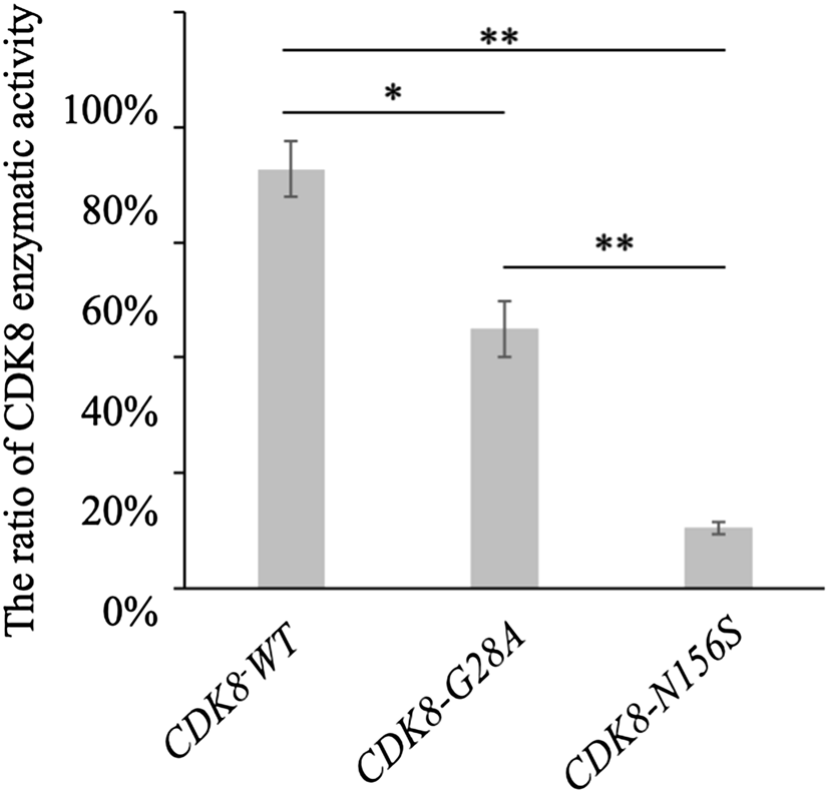
Results of human CDK8 kinase assay. The graph shows the enzymatic activity rates of wild-type CDK8, G28A-mutated CDK8, and N156S-mutated CDK8. The error bars indicate the standard error of the mean (SEM). *p < 0.05, **p < 0.005 using a t-test.

First, to evaluate the functional relevance of the p.G28A and p.N156S amino acid substitutions in human CDK8 in vitro, we performed a kinase assay using HEK293 cells, which are suitable for the analysis of exogenous protein expression. Both the *CDK8-G28A* and *CDK8-N156S* mutations resulted in lower kinase activities compared with *CDK8-WT* (Fig. 2), and the kinase activity of *CDK8-N156S* was lower than that of *CDK8-G28A* (Fig. 2). These results also indicated that the 2 types of mutated *CDK8* were hypomorphic alleles.

### In vivo functional assay of human CDK8 containing G28A and N156S substitutions

**Figure 3:**
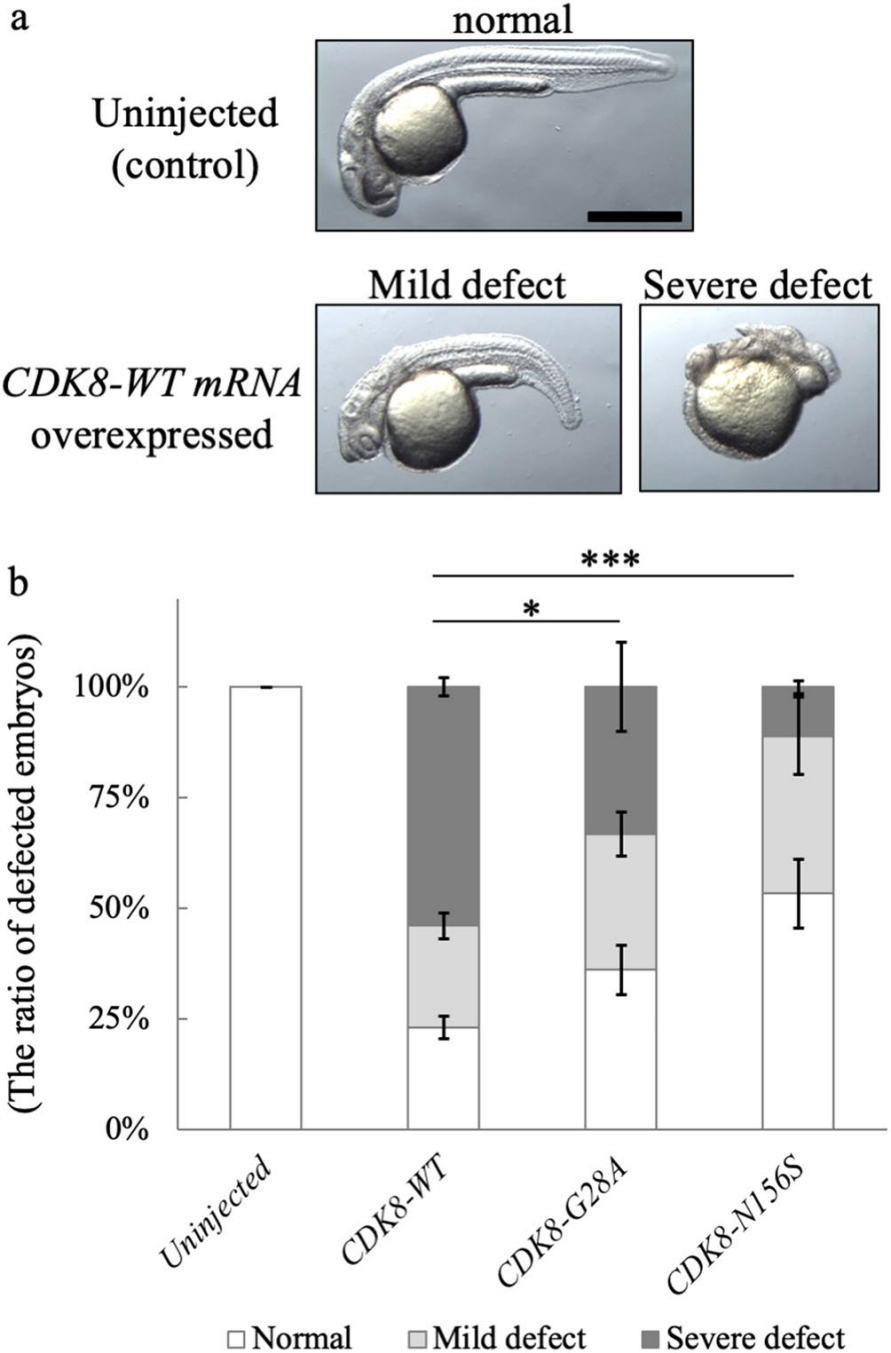
Results of *CDK8* mRNA overexpression analyses. (**a**) Photographs of zebrafish embryos at the 1-day post-fertilization (dpf) stage. An uninjected embryo was used as a control. The wild-type *CDK8* mRNA-injected embryo exhibited severe defects. Note that the *CDK8* mRNA injection induced a shortened body axis, small anterior structures, and an aberrant tail morphology. Scale bar, 500 μm. (**b**) Graph showing the proportions of abnormal zebrafish embryos. The error bars represent the SD for three independent experiments (for uninjected: n = 15, 36, 63 for CDK8: n = 34, 35, 31 for CDK8-G28A: n = 26, 65, 63 for CDK8-N156S: n = 27, 23, 28), *p = 0.02117, ***p = 6.224e−13 using a chi-square test.

Next, to evaluate the pathogenicity of the p.G28A and p.N156S amino-acid substitutions detected in Patient 1 and Patient 2, we injected zebrafish embryos with wild-type human CDK8 mRNA (*CDK8-WT* mRNA) and 2 types of mutated human CDK8 mRNA (i.e., *CDK8-G28A* mRNA and *CDK8-N156S* mRNA) and compared the zebrafish phenotypes at the 1-day post-fertilization (dpf) stage. Fraction of the embryos injected with *CDK8-WT* mRNA, *CDK8-G28A* mRNA, or *CDK8-N156S* mRNA exhibited a short body axis, hypoplastic anterior structures, and an aberrant tail (Fig. 3a).

The relative fraction of embryos with defects was largest when mRNA encoding the human wild-type CDK8 protein was injected (Fig. 3b). This observation indicated that CDK8-G28A and CDK8-N156S are hypomorphic alleles. In addition, consistent with the results of the in vitro kinase assays, the defect-inducing activity of CDK8-N156S was lower than that of CDK8-G28A (Fig. 3b), suggesting that the N156A mutation severely reduces CDK8 activity, while the G28A mutation mildly reduces the activity.

### Chemical inhibition of *Cdk8* in zebrafish

**Figure 4:**
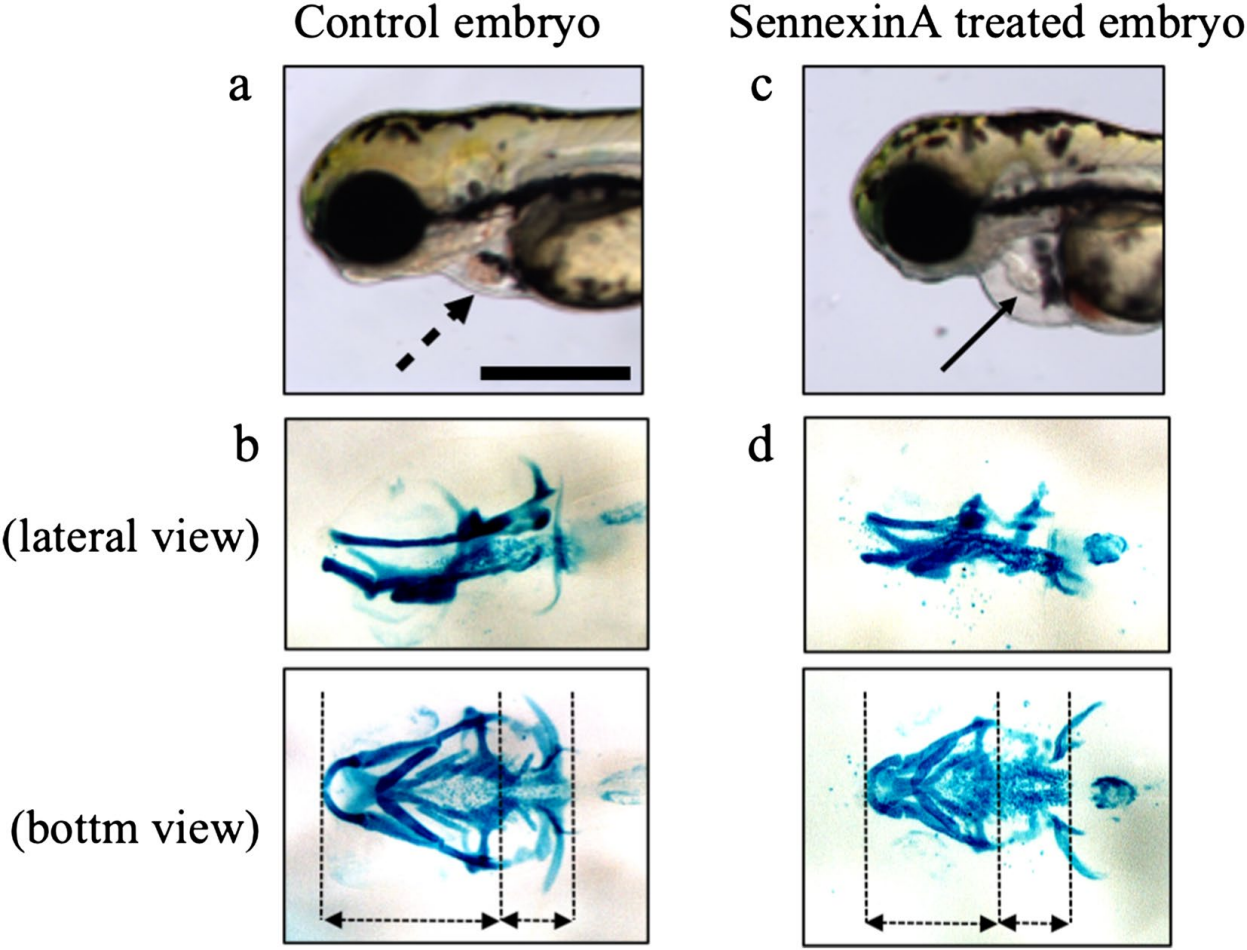
Results of chemical inhibition of Cdk8 in zebrafish. Photographs of the cranial cartilage of zebrafish embryos exposed to Senexin A at the 5 dpf stage. The upper photographs (**a**,**c**) are lateral view, and the lower photographs (**b**,**d**) are ventral view of the same embryo. (**a**,**b**) are photographs of a control embryo, and (**c**,**d**) are photographs of a Senexin A-exposed embryo. Note the cranial anomaly and shortened head in the fish. Scale bar in a, 0.2 mm.

We investigated whether the addition of Senexin A which inhibits CDK8 and its paralogous CDK19 would induce phenotypes reminiscent of the patients, and the results showed that the inhibition of Cdk8 kinase activity by Senexin A induced structural anomalies in the heart and craniofacial cartilage of the zebrafish (Fig. 4c,d, n = 46, 100%) compared to the control embryo (Fig. 4a,b) and disturbed blood circulation (Supplementary Video S1 and S2).

## Discussion

We showed herein that *CDK8*-associated disorder is caused by a reduction in CDK8 function using an in vivo functional assay, as supported by a previously reported in vitro assay^6^*.* We presented the cases of two patients with *CDK8*-associated disorder and suggested that craniofacial and cardiac defects are critical components of the newly identified disorder.

In this study, an in vivo functional study in a zebrafish model showed that the G28A and N156S mutations in the *CDK8* gene detected in the two presently reported patients were hypomorphic alleles (Fig. 3). We also showed that the in vitro kinase activities of CDK8-G28A and CDK8-N156S proteins, which were produced in human embryonic kidney (HEK293) cells, were significantly lower than that of the wild-type CDK8 protein (Fig. 2). These results agree with the observation of kidney defects in a patient with a CDK8 mutation. In addition, the results of our in vivo and in vitro assays were consistent with those of a previous study^6^*.*

The reduction of the CDK8 activity induced by N156S mutation was larger than that induced by G28A mutation. Furthermore, relative fraction of embryos with severe defects was larger after injection of mRNA encoding the G28A mutant proteins than after injection of mRNA encoding the N156S. Hence, both the in vitro kinase assays and the zebrafish in vivo assays showed that the N156S variant had a stronger effect than the G28A variant. This in vitro and in vivo results may be due to that N156 is more critical for CDK8 kinase activity, comparing with G28. Consistent with this idea, N156 is a highly conserved residue between CDK family kinases and locates on the T-loop, which is required for Cdk activation, while G28 is conserved between only a part of CDKs.

In humans and zebrafish, CDK8 has a paralog called CDK19 (also known as CDK8L), and CDK8 and CDK8L/CDK19 are generally assumed to have redundant roles. The current observation that the two patients with CDK8 mutations were symptomatic even in the presence of intact CDK19 suggests that CDK8 and CDK19 are not completely redundant from a functional standpoint. Furthermore, since mutant kinases lacking catalytic activity often confer a dominant-negative effect through interactions with their endogenous substrate and regulators, the mutant CDK8 protein might have interfered with the normal CDK19 protein.

Our observation that Senexin A, which inhibits CDK8, induced phenotypes reminiscent of those in the patients with CDK8 mutations supports the notion that CDK8 mutations confer a hypomorphic effect. This observation, however, needs to be interpreted with caution, since Senexin A inhibits both CDK19 and CDK8. At the same time, one could argue that the inhibitory action of Senexin A on both CDK19 and CDK8 may indeed mimic the presumable dominant-negative effect of the CDK8 mutations on CDK19 as well. Overall, the results of our in vivo analyses provide further credence to the notion that *CDK8*-associated disorder is caused by a functional reduction of CDK8, as suggested by the in vitro assay reported by Calpena et al.^6^.

In this study, we further defined the characteristic phenotypes of the newly identified disorder described in the initial report^6^. The characteristic features of our two patients and the 12 patients in the initial report^6^ are summarized in Table 1. Thirteen of the 14 patients had neurodevelopmental disabilities, and Patients 1 and 6 of the previously reported patients had congenital heart disorders. Common characteristic facial features include arched eyebrows (5/14), epicanthal folds (4/14), ptosis (6/14), anteverted nares (5/14), broad nasal root (5/14), and micrognathia (6/14). The neurodevelopmental abnormalities, congenital heart disorders, and facial features described above are characteristics of *CDK8*-associated developmental disorder. The phenotypes seem diverse, but the phenotypes of the *Cdk8*-knockdown zebrafish in the in vivo functional assay in our study were comparable to the phenotypes of patients with *CDK8*-associated disorder.

In conclusion, the results of this study suggest that *CDK8*-associated disorder is caused by the functional loss of CDK8 kinase activity. The phenotypes of the newly identified disorder are intellectual disability, characteristic facial features, and multiple organ defects. The phenotypes exhibited in the zebrafish model described above were comparable to the patients’ phenotypes.

## Methods

### Genetic analysis

Approval from the local institutional review board and informed consent from the patients’ parents were obtained prior to the molecular studies. DNA from peripheral blood samples obtained from the patients and their parents were isolated and subjected to exome sequencing with a SureSelect XT Human All Exon V6 Panel (Agilent Technology, Santa Clara, CA) on a HiSeq platform (Illumina, San Diego, CA). We confirmed the results by performing Sanger sequencing with the following primers: CDK8_Patient 1_sense, 5′-GCT CTT GCC GCA TCA GTC-3′; CDK8_Patient 1_antisense, 5′-GGC GTC GAG GAA GTC AGA G-3′; CDK8_Patient 2_sense, 5′-CAT GCA CAG TTT CTT TGG TGA C-3′; and CDK8_Patient 2_antisense, 5′-TGG CTT CGA TCA TAT GGA ACT AC-3′.

### In vivo functional assay

We performed in vivo functional analyses using a zebrafish model because zebrafish have a homologue of human CDK8, Cdk8. Cdk8 shares 96% homology with human CDK8 (Fig. 1a), and the sequences around the two reported variants are conserved in *Cdk8*. To determine whether the two variants reported in our patients were hypomorphic alleles, we compared the phenotypes of zebrafish injected with human *CDK8-WT* mRNA with those of zebrafish injected with mutated *CDK8* mRNA by performing the following experiment. First, we cloned the cDNA of wild-type human *CDK8* (a gift from Dr. Conaway, Addgene #15366)^10^ into the pCS2p + Flag vector in-frame of the Flag tag. The Gly28Ala (CDK8-G28A) and Asn152Ser (CDK8-N156S) point mutations identified in the patients’ DNA were then introduced into human CDK8 gene using a QuikChange Site-Directed Mutagenesis kit (Agilent). Next, Capped mRNA was synthesized using a SP6 mMESSAGE mMACHINE kit (Ambion, Austin, TX). The cDNA of wild-type human *CDK8* (a gift from Dr. Conaway, Addgene #15366)^10^ Capped mRNA was synthesized using an SP6 mMESSAGE mMACHINE kit (Ambion, Austin, TX) and purified on Micro Bio-Spin columns (Bio-Rad, Hercules, CA). We injected each of the synthesized mRNAs *(CDK8-WT*: n = 34, 35, 31*, CDK8-G28A*: n = 26, 65, 63, and *CDK8-N156S*: n = 27, 23, 28) into one-cell stage embryos (150 pg) and examined the morphological features at the 1-day-post-fertilization (dpf) stage of the embryos. The proportion of the phenotypes was determined by pooling the data from all three replicates and conducting a Chi-square test for *CDK8-G28A* or *CDK8-N156S* against *CDK8-WT*.

### Kinase assay

Flag-CDK8 and its mutants were expressed with CyclinC in HEK293 cells and immunoprecipitated with anti-Flag antibody. Immunoprecipitates were purified by washing six times with PBS. Aliquots of immunoprecipitated CDK8 proteins were incubated for 90 min with or without 50 μM ATP in 40 μL of kinase buffer containing 40 mM Tris (pH7.5), 100 μM BSA, and 20 mM MgCl2 at 25 °C, and the ADP produced by CDK8 autophosphorylation was measured using a ADP-GLO Kinase Assay kit (Promega). Immunoprecipitates from cells transfected with empty vector was used for measurement of background activity. The values shown are the average of the values observed in one representative experiment in which each transfection was performed in triplicate.

### Chemical inhibition of Cdk8 activity by Senexin A

The phenotypes of Cdk8 inhibition were investigated in zebrafish by treatment with the Cdk8/Cdk19-specific inhibitor Senexin A (2 μM) from 18 dpf. We then compared the phenotypes of the Senexin A-exposed zebrafish and the control zebrafish.

### Cartilage staining

To observe head formation, we performed cartilage staining using the following method. At 6 days post-fertilization, the embryos were fixed overnight in 4% paraformaldehyde/PBS. They were then dehydrated with ethanol and stained with 0.02% Alcian blue in an acid solution (40% acetic acid, 60% ethanol). After rehydration, the embryos were cleared in 2% KOH, bleached in 3% H_2_O_2_, and further cleared in glycerol.

### Ethics statement

All studies in this paper were approved by the local Institutional Review Board in Keio University School of Medicine, Ethics Committee, Gunma University, and Osaka University. All human studies were performed in accordance with the ethical standards of the Declaration of Helsinki. All experimental animal care was performed in accordance with institutional and national guidelines and regulations. The study protocol was approved by the Institutional Animal Care and Use Committee of the respective universities (Gunma University Permit# 17-051; Osaka University, RIMD Permit#R02-04-0).

## Supplementary information

Supplementary Video 1.

Supplementary Video 2.

Supplementary Legend.
