# Supplementary material for: Pathogenesis of CDK8-associated disorder: two patients with novel CDK8 variants and in vitro and in vivo functional analyses of the variants
Source: Sci Rep. 2020 Oct 16;10:17575. doi: 10.1038/s41598-020-74642-4 (PMC7567849; doi:10.1038/s41598-020-74642-4)
Supplement: Supplementary file 3 — Supplementary Legend. [file 41598_2020_74642_MOESM3_ESM.docx]

**Pathogenesis of *CDK8-*associated disorder: two patients with novel *CDK8* variants and *in vitro* and *in vivo* functional analyses of the variants**

Tomoko Uehara^1^, Kota Abe^2,6^, Masayuki Oginuma^2,6^, Shizuka Ishitani^2,6^, Hiroshi Yoshihashi^3^, Nobuhiko Okamoto^4^, Toshiki Takenouchi^5^, Kenjiro Kosaki^1^, Tohru Ishitani^2,6^

^1^ Center for Medical Genetics, Keio University Hospital, Tokyo, Japan

^2^ Institute for Molecular & Cellular Regulation, Gunma University, Maebashi, Japan

^3^ Department of Genetics, Tokyo Metropolitan Children’s Medical Center, Tokyo, Japan

^4^ Department of Medical Genetics, Osaka Women’s and Children’s Hospital, Osaka, Japan

^5^ Department of Pediatrics, Keio University Hospital, Tokyo, Japan

^6^ Department of Homeostatic Regulation, Research Institute for Microbial Diseases, Osaka University, Osaka, Japan

Correspondence to: Tohru Ishitani, PhD

Research Institute for Microbial Diseases, Osaka University

3-1 Yamadaoka, Suita, Osaka 565-0871, Japan

Tel: +81-6-6879-8358; E-mail: ishitani@biken.osaka-u.ac.jp

**Supplementary video. Heart defect arising from the chemical inhibition of Cdk8 in zebrafish.**

Movies of the hearts of zebrafish embryos exposed to Senexin A at the 3 dpf stage. S1 is a control embryo, and S2 is a Senexin A-exposed embryo. The Cdk8-inhibited embryo had an abnormal heart morphology and beating, causing a defect in blood circulation.
